# Sticky Multicolor Mechanochromic Labels

**DOI:** 10.1021/acsami.3c19420

**Published:** 2024-03-06

**Authors:** Lucas D. C. de Castro, Tom A. P. Engels, Osvaldo N. Oliveira, Albert P. H. J. Schenning

**Affiliations:** † São Carlos Institute of Physics, University of São Paulo, São Carlos 13566-590, SP, Brazil; ‡ Laboratory of Stimuli-responsive Functional Materials and Devices (SFD), Department of Chemical Engineering and Chemistry, Eindhoven University of Technology, Eindhoven5612 MB, The Netherlands; § Processing and Performance of Materials, Department of Mechanical Engineering, Eindhoven University of Technology, Eindhoven 5600 MB, The Netherlands; ∥ Institute for Complex Molecular Systems, Eindhoven University of Technology, Eindhoven 5612 MB, The Netherlands

**Keywords:** mechanochromic devices, cholesteric liquid crystal elastomers, stickers, labels, wearable devices

## Abstract

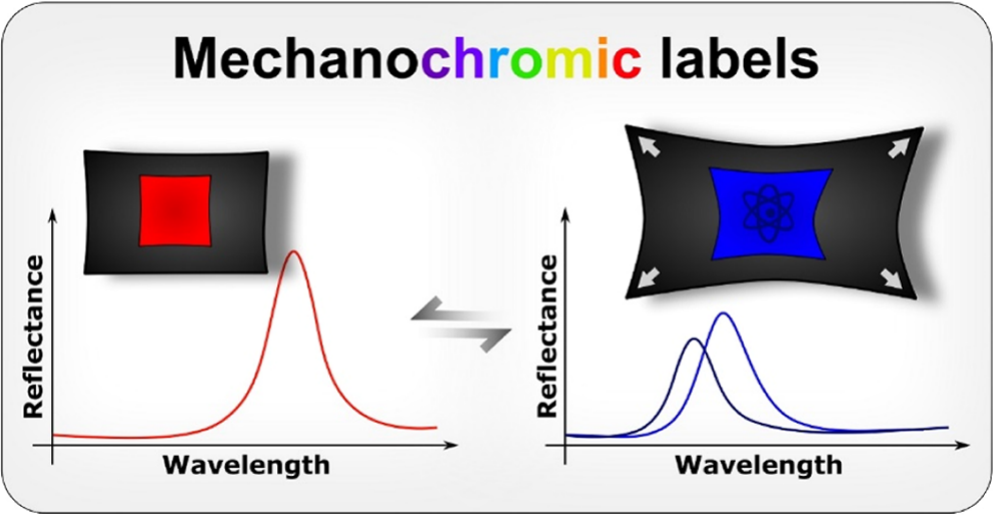

Sticky-colored labels are an efficient way to communicate visual information. However, most labels are static. Here, we propose a new category of dynamic sticky labels that change structural colors when stretched. The sticky mechanochromic labels can be pasted on flexible surfaces such as fabric and rubber or even on brittle materials. To enhance their applicability, we demonstrate a simple method for imprinting structural color patterns that are either always visible or reversibly revealed or concealed upon mechanical deformation. The mechanochromic patterns are imprinted with a photomask during the ultraviolet (UV) cross-linking of acrylate-terminated cholesteric liquid crystal oligomers in a single step at room temperature. The photomask locally controls the cross-linking degree and volumetric response of the cholesteric liquid crystal elastomers (CLCEs). A nonuniform thickness change induced by the Poisson’s ratio contrast between the pattern and the surrounding background might lead to a color-separation effect. Our sticky multicolor mechanochromic labels may be utilized in stress–strain sensing, building environments, smart clothing, security labels, and decoration.

## 1 Introduction

Dynamic color control enables communication and interaction between living species in a diversity of biological and artificial environments.^1−4^ This has been the inspiration for the development of mechanochromic materials whose structural color changes in response to mechanical stimuli.^5−7^ Cholesteric liquid crystal elastomers (CLCEs) are prominent among such materials as the structural colors can be easily programmed. The colors arise from the helical twisting of anisotropic rod-shaped liquid crystal (LC) molecules along an axis perpendicular to the molecular director which in turn can be tuned by the concentration of the chiral dopant.^8−12^ The programmable dynamic structural colors of CLCEs make them prominent candidates for information delivery.^13−17^ However, the mechanochromic effect is usually manifested by translucent thin films,^18−20^ thus integration with real-world applications is challenging. Moreover, mechanochromic materials are normally limited to uniform color-shifting which restrains their communication capabilities.^21−24^ Visual information for ornamentation or functional purposes can be conveyed with sticky labels. These labels typically comprise printed material with an adhesive layer on the back side that can be attached to different surfaces. Since sticky labels can be customized and printed with arbitrary information, they are useful for decoration, identification, branding, pricing, safety, and advertising. Nonetheless, most of the sticky labels are static, which limits their information transfer.

In this paper, we present a new category of dynamic CLCE sticky multicolor mechanochromic labels to enhance their applicability. As a desired feature for any label, we demonstrate a method for imprinting color patterns that are either always visible or reversibly revealed or concealed upon mechanical deformation. CLCE patterning is complex and often involves multiple steps combined with temperature,^18,21^ incorporation of nanoparticles,^25,26^ chemical modification,^27,28^ external electronic devices,^29,30^ and consecutive photocuring and rinsing steps.^31^ Toward a much simpler approach, our patterns are imprinted during the ultraviolet (UV) cross-linking in a single step at room temperature. This is achieved through the localized control of the cross-linking density with a photomask. The cross-link density apparently influences the volumetric response (i.e., the Poisson’s ratio) of the CLCE, thus nonuniform thickness changes to external deformation can be locally programmed and manifested macroscopically in a blue shift mismatch between the pattern and background. More than simply expressing words or visual images, these sticky mechanochromic multicolor labels can be useful in complex stress–strain sensing, monitoring of structural integrity, smart clothing, security labels, and decoration while also offering unprecedented opportunities for the fashion industry and art design.

## 2 Experimental Section

### 2.1 Materials

The diacrylate mesogens 2-methyl-1,4-phenylene bis(4-((6-(acryloyloxy)hexyl)oxy)benzoate) (RM82) and 2-methyl-1,4-phenylene bis(4-(3-(acryloyloxy)propoxy)benzoate) (RM257) were purchased from Daken Chemical. The chiral dopant ((3R,3aS,6aS)-hexahydrofuro[3,2-*b*] furan-3,6-diyl bis(4-(4-((4-(acryloyloxy)butoxy)carbonyloxy)benzoyloxy)benzoate)) (LC756) was purchased from BASF.; 2,2′-(ethylenedioxy)diethanethiol (EDDET); dipropylamine (DPA) and poly(vinyl alcohol) (PVA, *M*_w_ 31,000–50,000) were purchased from Sigma-Aldrich. Irgacure 651 was purchased from Ciba Specialty Chemicals. Dichloromethane (DCM) was purchased from Bio Solve. RTV-1 silicone (Elastosil E43) was purchased from Wacker.

### 2.2 Preparation of the CLCE Oligomer Ink

The diacrylate mesogens RM82 (1.040 mMol); RM257 (0.340 mMol); chiral dopant LC765 (0.057 mMol); and dithiol monomer EDDET (0.691 mMol) were added to a glass vial and dissolved in 5 mL of DCM. DPA (10 μL) was added as a catalyst for the first-stage Michael addition reaction. The glass vial was closed tightly with a lid, and the solution was stirred overnight at room temperature to obtain an acrylate-terminated CLC oligomer. Then, Irgacure 651 (0.043 mMol) was added to the solution and the oligomer was dried overnight in a vacuum oven at 60 °C. The molecular structures of the chemicals employed in the oligomer synthesis are shown in Figure S1.

### 2.3 Preparation of Sticky Mechanochromic Labels

The labels were prepared onto a poly(ethylene terephthalate) (PET) film substrate via wire bar coating using an RK K control coater. Prior to the coating process, the PET strip is rinsed with acetone and dried with nitrogen. The top edge of a strip is fixed to the bar-coater table, which is set to 70 °C. A wire bar with a 4 μm gap is placed in the holder, and a 10 wt % PVA aqueous solution is deposited onto the substrate as a sacrificial layer. A wire bar with a 16 μm gap is placed in the holder, and the as prepared acrylate-terminated CLC oligomer ink is deposited onto the PET/PVA layer. Then, a UV cross-linking fixed on 5 s was performed with an Omnicure series 2000 EXFO lamp under nitrogen (N_2_) atmosphere. Color patterns were created by using grayscale photomasks. The photomasks were prepared on a transparent foil using a regular laser printer and placed between the sample and the UV light source. The UV doses are dictated by the gray levels and measured with a RM12 from Opsytec Dr. Grobel instrument equipped with a UV-A (400–315 nm) sensor. After UV cross-linking, the samples were cut into the desired shape/size.

### 2.4 Label Application

A thin layer of a retail RTV-1 silicone (Elastosil E43) was manually spread on the label and gently pressed against the target stretchable substrates. The silicone is solvent-free and cures at room temperature under the influence of atmospheric moisture. After a few minutes, the system was immersed in water to dissolve the sacrificial PVA layer, and the PET substrate was peeled off to reveal the mechanochromic labels attached to the target substrate.

### 2.5 Material Characterization

^1^H-nuclear magnetic resonance (^1^H NMR) measurements were performed on a Bruker Avance Core III 400 MHz spectrometer using deuterated chloroform as the solvent; the average length was calculated using the relative integrals of the remaining acrylate groups and the aromatic hydrogens of the liquid crystal units.^32^ The glass transition (*T*_g_) and cholesteric to isotropic transition (*T*_Ch,I_) temperatures were determined by differential scanning calorimetry (DSC) with a TA Instruments Q2000 apparatus; samples were placed on aluminum hermetic crucibles and scanned during heating and cooling cycles from −50 to 150 °C at a 10 °C/min rate. The transition temperatures were obtained from the second and third heating cycles. Fourier-transform infrared (FTIR) measurements were performed with a Varian 670 FTIR spectrometer with a slide-on ATR (Ge). Optical images of the mechanochromic labels were taken with a Leica DM2700 M polarized optical microscope (POM) operating in reflection and transmission mode. Reflectance spectra were measured using a fiber-coupled Ocean Optics HR2000+ spectrometer equipped with a POM microscope; a 20× lens with a numerical aperture of 0.40 was used for spectra acquisition.

## 3 Results and Discussion

### 3.1 Preparation of Sticky Mechanochromic Labels

**Figure 1:**
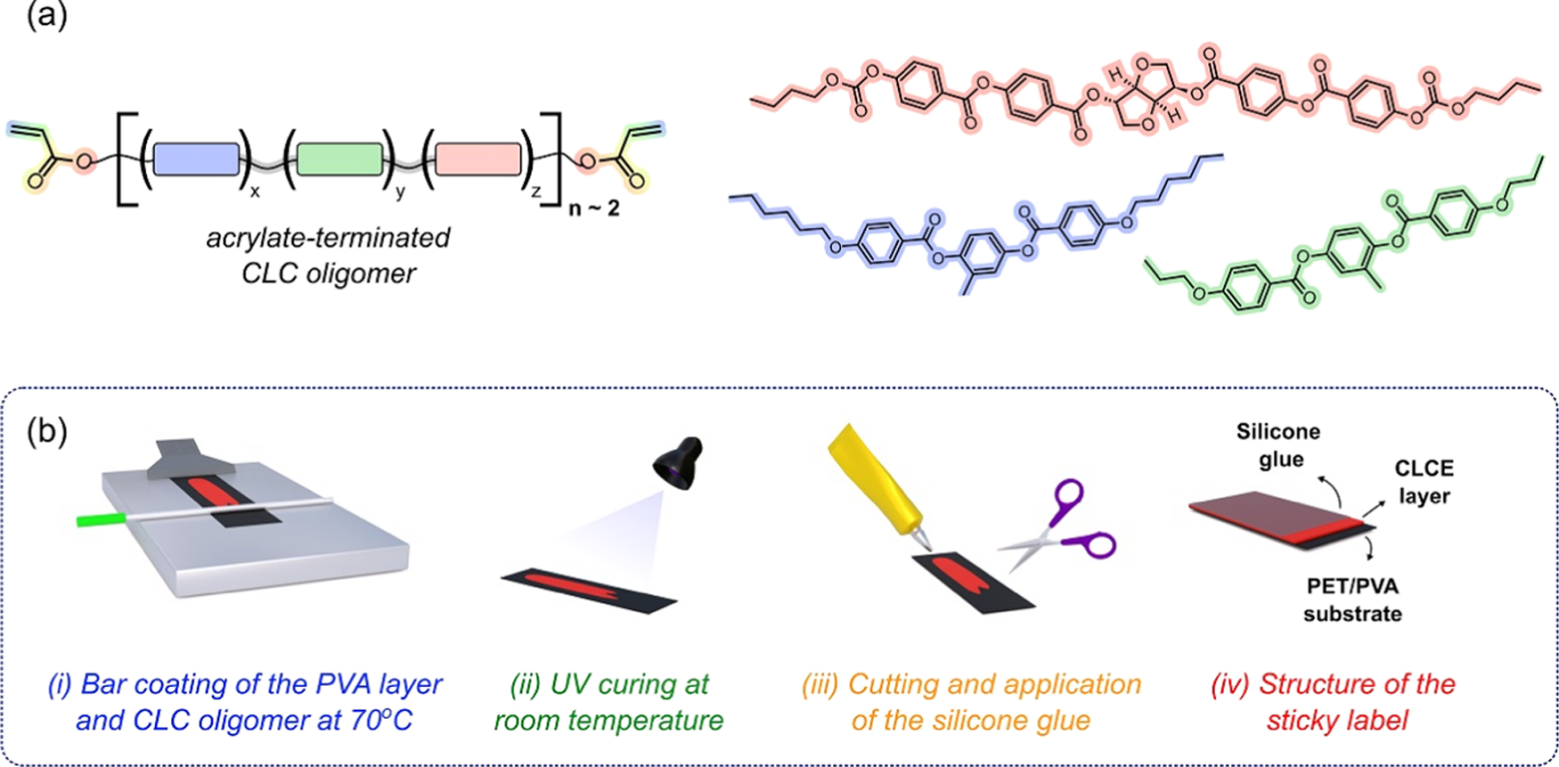
(a) Chemical structure of the acrylate-terminated CLC oligomer and (b) preparation of the sticky multicolor mechanochromic labels from the CLC oligomer ink.

The sticky mechanochromic labels comprise a CLCE deposited on a water-soluble PVA sacrificial layer supported by a PET film substrate. CLCE is based on an acrylate-terminated oligomeric precursor synthesized following a thiol–acrylate Michael addition reaction. Dipropylamine was used as a catalyst to initiate the click reaction between two diacrylate mesogens (RM82 and RM257), a diacrylate chiral dopant (LC756), and a dithiol chain extender (EDDET). By controlling the molar ratio of acrylates and thiols, an acrylate-terminated oligomer with average length of 2 monomer units was synthesized (Figure 1a). A short oligomer was selected to facilitate processability without using solvents. The concentration of chiral dopant was adjusted to obtain a red-colored label. The ^1^H NMR spectra (Figure S2) of the CLC oligomers revealed a degree of polymerization (DP) of 2.1 by using the relative integrals of the remaining acrylate groups and the aromatic hydrogens of the liquid crystal units. From the DSC analysis (Figure S3), we determined a glass transition temperature (*T*_g_) of −27 °C and a cholesteric to isotropic phase transition temperature (T_Ch,I_) of 85 °C.

A schematic illustration of the preparation of mechanochromic sticky labels is shown in Figure 1b. A sacrificial layer of PVA was applied to a PET film and the CLC oligomer mixed with photoinitiator was bar-coated without the need of a solvent on the PET/PVA substrate at 70 °C. This temperature was selected because it is just below *T*_Ch,I_ where the viscosity is lower.^33−35^ After the bar coating, the oligomer ink was UV cross-linked with a dose of 4 mJ/cm^2^ under N_2_ atmosphere at room temperature. A colored film was obtained, and the POM micrographs evidence a shear-induced planar alignment of CLCE (Figure S4). From the DSC thermogram, no discernible peak indicating a cholesteric to isotropic transition (*T*_Ch,I_) could be identified. However, this transition can be observed by polarized optical microscopy at around 120 °C. The FTIR spectrum indicates, although diminished, the presence of acrylate groups pointing to a partially cross-linked network (Figures S5 and S6). The labels were cut in the desired shape/size and the silicone glue was manually spread on their surface before use. A silicone glue was selected because it provides mechanical robustness, cures at room temperature under the influence of atmospheric moisture, and has good adhesion to many substrates.

**Figure 2:**
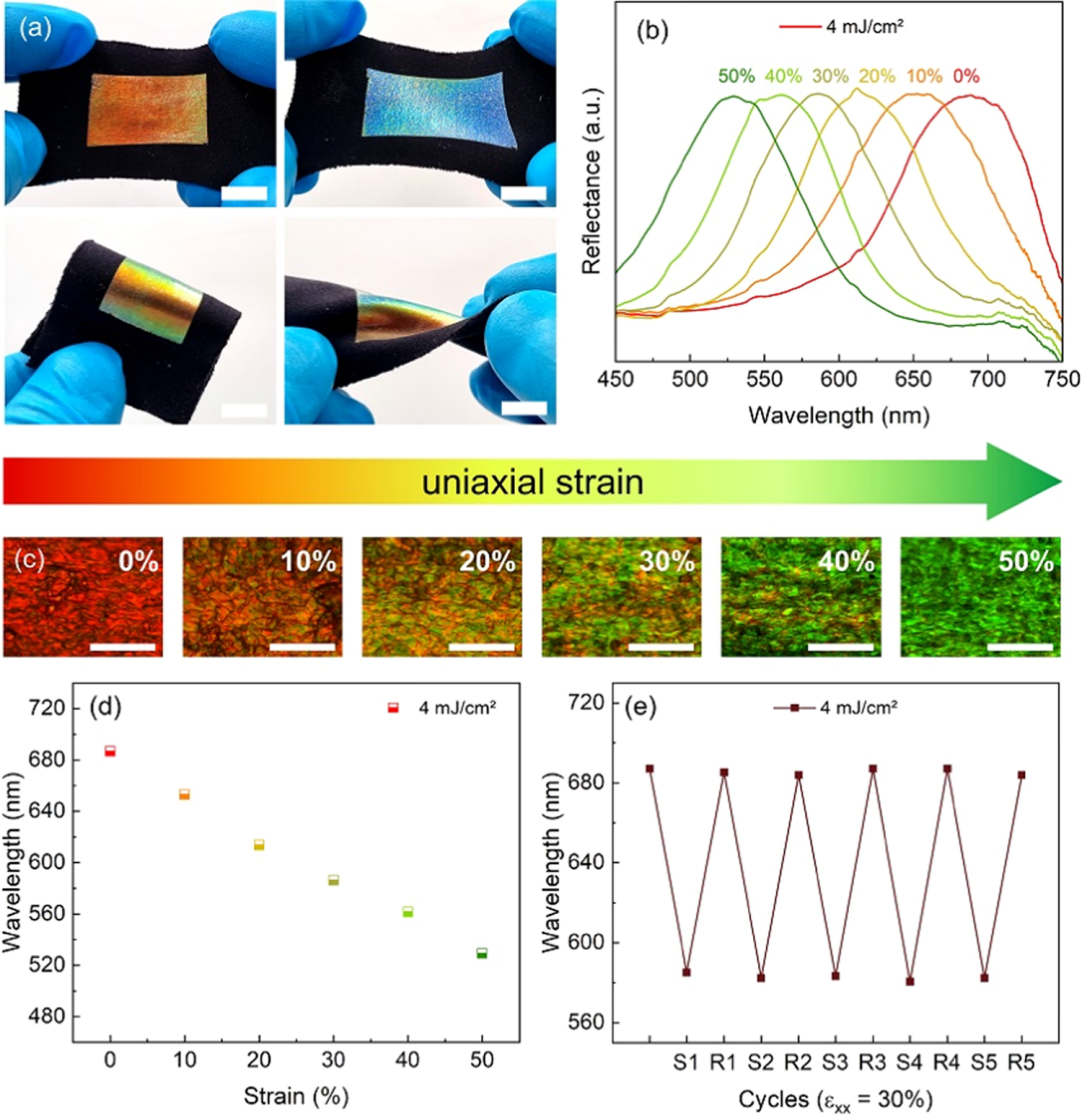
Optical properties of the mechanochromic label UV cross-linked with 4 mJ/cm^2^. (a) Photographs of the labeled fabric (upper left) in the relaxed state; (upper right) under nonuniform strain; (lower left) bent; and (lower right) twisted (scale bars are 1 cm). (b) Reflectance spectra of the label under uniaxial strain and (c) their corresponding optical micrographs (scale bars are 25 μm). (d) Wavelength shift of the label obtained from the reflectance curves. (e) Reversible color-changing behavior of the label submitted to stretching-releasing cycles (ε_*xx*_ = 30%). S1 represents the first stretched state, and R1 represents the first relaxed state.

For characterization, the label is gently pressed against a piece of stretchable black fabric and immersed in water for 1 h to peel off the PET/PVA supporting layer. After peeling, a red label is obtained. From the cross-section of the labeled fabric shown in Figure S7, the thickness of the substrate, silicone glue and CLCE are respectively around 750 μm, 40 μm, and 16 μm. The label exhibits a mechanochromic behavior showing a color change to green and blue when stretched. The structural colors are angle-dependent owing to iridescence arising from the aligned CLC oligomer. Hence, a rainbow-like effect is observed when the label is twisted or bent (Figure 2a). The periodicity of the helical pitch (*p*) determines the central reflection wavelength (λ) according to the Bragg’s law λ = *n*.*p*.cos θ, where n is the average refractive index of the LCs molecules, and θ is the angle of incident light.^36^ To investigate the mechanochromic behavior in detail, the labeled textile was clamped and stretched. The reflectance spectra and optical micrographs of the label recorded under uniaxial strain are shown in Figure 2b–d. During mechanical deformation, the structural colors blue shift in a reversible manner. When the label is stretched to 50% the wavelength of the label shifted from 687 to 529 nm and the relative color spectral shift (Δλ/λ_0_) was 23%, which is comparable to other reported structural colored systems.^21,27,31^ The spectral shift is maintained during sequential stretching and releasing cycles, pointing to a stable and fully reversible mechanochromic effect (Figure 2e). The labels exhibited mechanical robustness compatible with daily activities (Movie S1) and maintained their mechanochromic behavior for at least several months. It should be noted that the fine control of UV dose during photopolymerization is important for obtaining partially cross-linked CLCEs with reversible mechanocromism. In the case of a fully cross-linked sample, stretching leads to cracks, while for a non-cross-linked sample, the application on a target surface is not even possible (Figure S8 and Movie S2).

**Figure 3:**
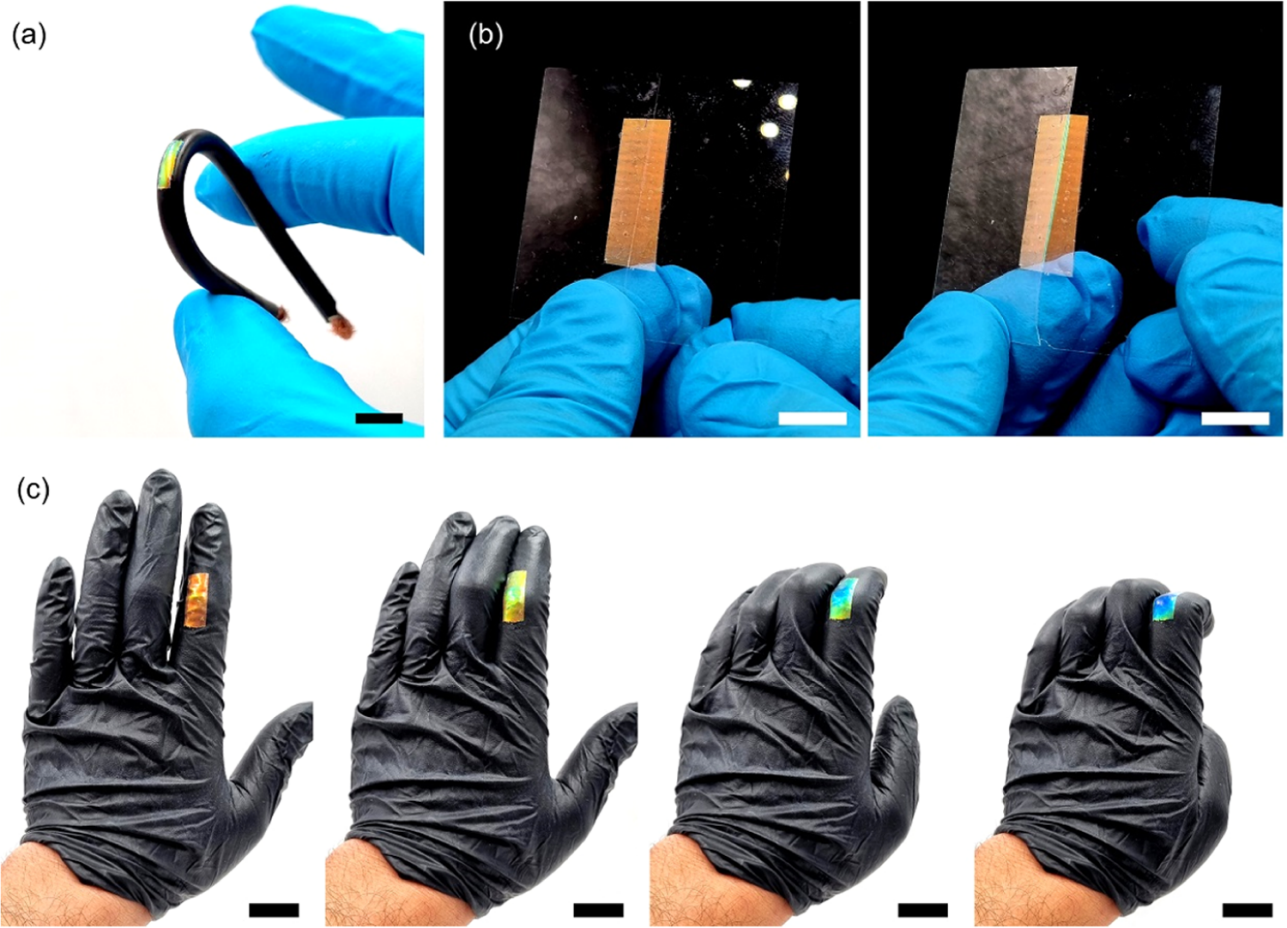
Examples of applications of sticky mechanochromic labels. (a) Electricity cable. The label can indicate if the cable is excessively deformed (scale bar is 1 cm). (b) Evaluation of structural integrity and crack propagation of brittle materials. The stress-concentrator effect locally blue shifts the red label when a stable crack (left) becomes unstable and starts to propagate (right), indicating the crack path in a strong green color (scale bars are 1 cm). (c) Strain sensor for monitoring human mobility. The red label was applied on a latex glove and reversibly blueshifts during bending of the finger (scale bars are 2 cm).

Sticky mechanochromic labels can be employed on different substrates. For example, the label can be pasted on cables to indicate excessive deformation (Figure 3a). In a building, the label can evaluate structural integrity and crack propagation in brittle materials such as glass. If a stable crack becomes unstable and starts to propagate, the stress-concentration effect induces a local blue shift on the red label, and the crack path immediately becomes visible as the color changes to green (Figure 3b). When applied on a garment such as a latex glove, the label can be used as a strain sensor to monitor human mobility. During the bending of the finger, the structural color blue shifts from red to blue in a reversible manner. The color tone can be used to estimate the amplitude of the motion (Figure 3c and Movie S3).

### 3.2 Multicolor Mechanochromic Labels

**Figure 4:**
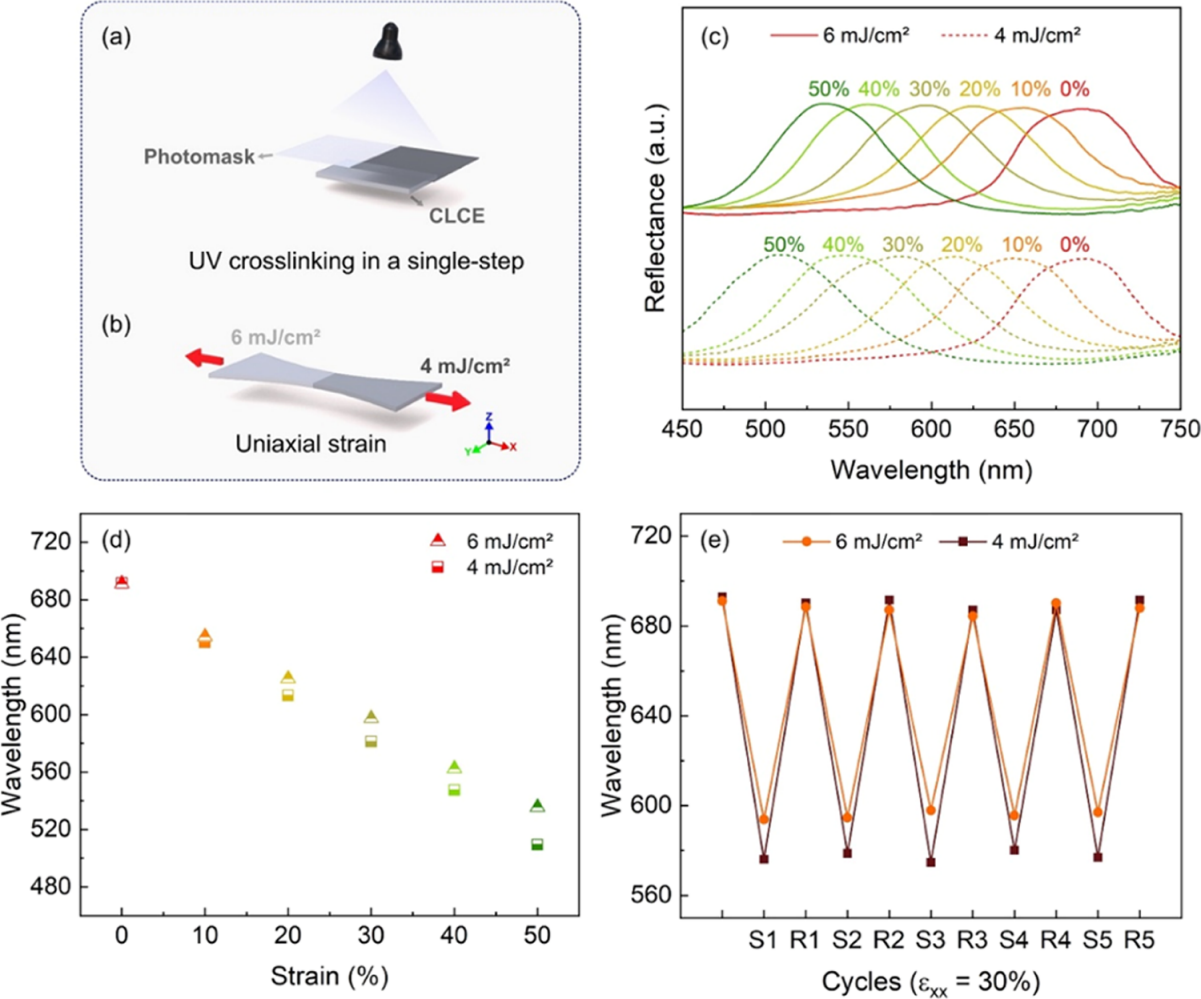
Color-separation mechanism of the label UV cross-linked with 6 and 4 mJ/cm^2^. (a) UV cross-linking of the label. A photomask was prepared with a rectangular pattern divided into two halves that exhibit ca. 50% of transmittance difference. The UV dose was adjusted to 6 and 4 mJ/cm^2^ in the clear and dark regions, respectively. The photomask was placed between the light source and the sample during UV cross-linking which was performed in a single step. (b) Schematic illustration of the uniaxial strain applied on the samples. (c) Reflectance spectra of the 6 and 4 mJ/cm^2^ halves recorded under uniaxial strain. (d) Mismatch of the spectral shift between the 6 and 4 mJ/cm^2^ halves. (e) Reversible color-changing behavior of the mechanochromic label submitted to stretching-releasing cycles. S1 represents the first stretched state and R1 represents the first relaxed state.

The utility of the sticky labels can be extended by creating tailored mechanochromic patterns through controlling the cross-linking degree by changing the UV dose. The UV dose can be adjusted with grayscale photomasks. We fabricated a photomask on a transparent polyester film by using a standard laser printer. The photomask contains a grayscale rectangular pattern divided into two parts that exhibit a ca. 50% transmittance difference. Here, the UV doses were adjusted to 6 and 4 mJ/cm^2^ in the clear and gray regions, respectively. After a single UV cross-linking step using the grayscale mask (Figure 4a), the label was pasted on the stretchable black fabric. In the relaxed state, both halves exhibit λ = 691 nm, and the pattern is invisible. Then the labeled textile was submitted to a controlled uniaxial deformation as in Figure 4b, and the reflection spectra of the two halves were recorded. As the bipartite label is stretched, the reflection spectra of both regions blue-shifted differently, and a gradual color separation was observed (Figure 4c,d). For instance, when the labeled fabric is stretched to 50%, the 6 and the 4 mJ/cm^2^ halves blue-shifted to 536 and 509 nm, respectively; in this case, the color spectral shifts are 22 and 26%. As the CLCE thickness is much larger than the helical pitch, the relative color spectral shift (Δλ/λ_0_) is equal to |ε_*zz*_|.^37^ The in-plane deformation (ε_*xx*_, ε_*yy*_) is dictated by the thick fabric substrate and is the same for the whole CLCE film, hence the changes in thickness of the 6 mJ/cm^2^ and the 4 mJ/cm^2^ halves might be different. This difference in thickness, while the lateral dimensions are assumed to be the same, brings forward a difference in volume change of the two halves, and thus their Poisson’s ratios are different. It seems therefore reasonable to suggest that the cross-link density influences the Poisson’s ratio of the CLCE and that, at an applied strain, the local thickness reduction of the 6 mJ/cm^2^ region is lower than that of the 4 mJ/cm^2^ region, inducing a difference in the spectral shift. When the stress is released, both halves return to the initial state, and a uniform color is observed. Figure 4e shows that the spectral shift consistency is preserved in stretching and recovering cycles owing to the elastic behavior of the stickers.

**Figure 5:**
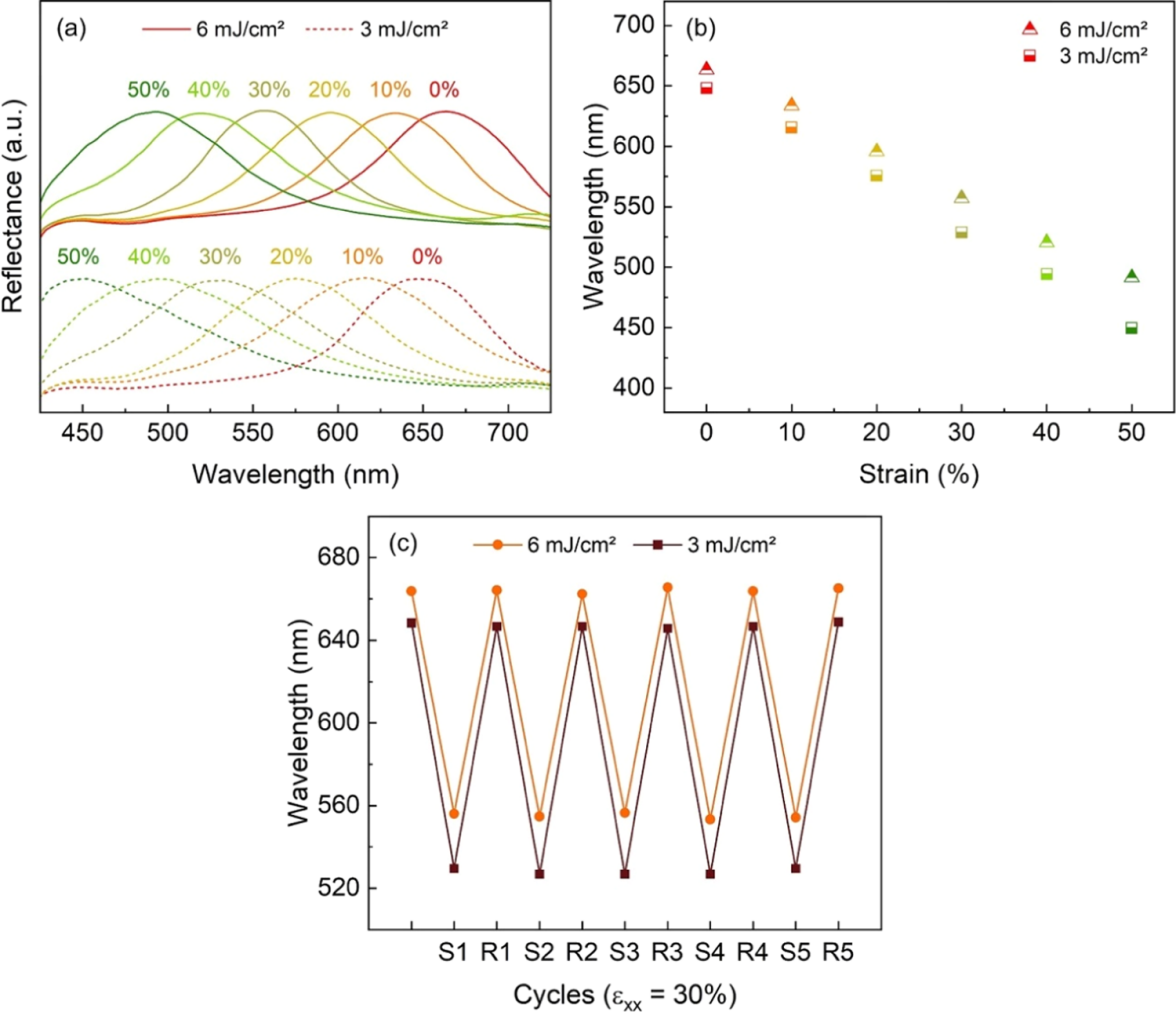
Color-separation mechanism of the label UV cross-linked with 6 and 3 mJ/cm^2^. (a) Reflectance spectra of the 6 and 3 mJ/cm^2^ halves recorded under uniaxial strain. (b) Mismatch of the spectral shift between the 6 and 3 mJ/cm^2^ halves. The inset illustrates the uniaxial deformation applied in the iso-stress condition. (c) Reversible color-changing behavior of the label submitted to stretching-releasing cycles. S1 represents the first stretched state and R1 represents the first relaxed state.

The role of UV dose on the patterning of CLCE labels was studied further by preparing another photomask containing the same rectangular geometry divided into two parts with increased transmittance difference. The transmitted UV doses were now adjusted to 6 and 3 mJ/cm^2^ in the clear and gray regions, respectively. Analogously to the previous example, the label was applied on a stretch fabric after the UV cross-linking in a single step. The labeled textile was clamped, and the reflectance spectra of both halves were recorded during the controlled uniaxial deformation (Figure 5a,b). Interestingly, a mismatch of 15 nm between the reflectance spectra was already observed in the rest of the state revealing a bicolored pattern. When ε_*xx*_ = 0%, the λ of 6 and 3 mJ/cm^2^ are 663 and 648 nm, respectively. When compared to the first example, the initial color of the 6 mJ/cm^2^ region might be due to batch-to-batch difference, leading to small changes in the concentration of the chiral dopant in the ink. The blue-shifted color at 3 mJ/cm^2^ UV dose might be due to the changes in the helical twisting power of the chiral dopant during the slow polymerization kinetics.^38,39^ Moreover, as the bicolored label was stretched, the reflection spectra blue-shifted nonuniformly, and the color-separation effect was enhanced. The color-separating effect can again be attributed to the nonuniform thickness change between the 6 and 3 mJ/cm^2^ regions. Here, when the labeled fabric was stretched to 50%, the 6 mJ/cm and the 3 mJ/cm^2^ halves respectively blue-shifted to 491 and 450 nm, leading to Δλ/λ_0_ of 26 and 31%. Since the Δλ/λ_0_ of the 3 mJ/cm^2^ half is considerably higher than its counterpart, one further infers that some degree of molecular reorganization occurred. The molecular mobility is evidenced by the presence of a small *T*_Ch,I_ peak on the DSC thermogram of a sample UV cross-linked with to 3 mJ/cm^2^ (Figure S9). Despite this molecular mobility during stretching, the label was anchored by a highly elastic substrate, which ensured a reversible behavior. Thus, both halves returned to their initial state when the stress was released, as in Figure 5c.

**Figure 6:**
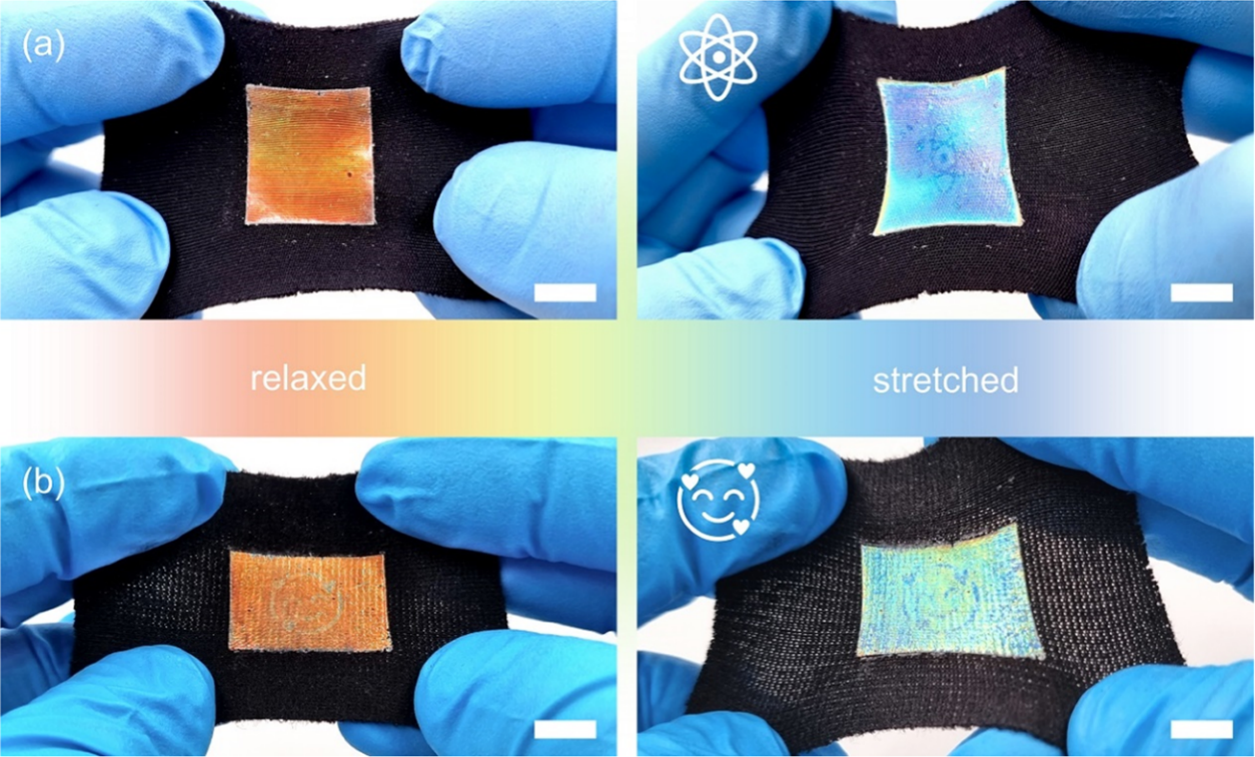
Macroscopic response of the color-separating patterns. (a) Atom icon. The atom icon is revealed upon deformation and concealed in the relaxed state. (b) Emoji icon. The emoji icon is always visible and becomes clearer upon deformation (scale bars are 1 cm).

The previous findings were used to prepare sticky, multicolored mechanochromic labels for different fabrics. To explore the control of the degree of cross-linking, we prepared photomasks with UV doses fixed at 6 mJ/cm^2^ for the background and varied the transmittance of the pattern. In the first example, we prepared an invisible atom icon that is revealed and concealed upon mechanical deformation by adjusting the UV dose of the pattern to 4 mJ/cm^2^. The sticky label was then applied to a black Spandex fabric and stretched. In this case, we induced a lower cross-linking degree in the atom icon, creating a Poisson’s ratio contrast with the surrounding background. In the relaxed state, the reflection band is uniform along the entire CLCE sticker; thus, the atom icon is invisible. Upon stretching, the Poisson’s ratio difference is responsible for a nonuniform thickness change, and a mismatch on the reflection spectra blue shift is observed. The atom icon is then instantly revealed by the difference in the mechanochromic response of the two regions. When the strain is released, the mechanochromic label goes through a reversible red shift and returns to its initial state, making the atom icon invisible again (Figure 6a and Movie S4). In a second example, we prepared an emoji icon that is already visible in the undeformed rest state by adjusting the UV dose of the emoji pattern to 3 mJ/cm^2^. Now, the label was applied on Viscolycra fabric, which exhibits a less uniform texture and less stretchability than the Spandex fabric. Analogous to the first example, the Poisson’s ratio difference also results in a nonuniform thickness change upon mechanical deformation. Therefore, Δλ between the pattern and the background is further enhanced during stretching, making the emoji ion even more clear. When the strain is released, the system returns to its initial state and the emoji icon is still visible (Figure 6b and Movie S5).

## 4 Conclusions

We developed sticky mechanochromic labels that change structural colors dynamically when stretched. The labels can be pasted onto different substrates. To enhance their information delivery capability, we also demonstrated a method to imprint color patterns that are either always visible or reversibly revealed or concealed upon mechanical deformation. In a simple approach, the patterns were imprinted during the UV cross-linking in a single step at room temperature. This was achieved through the localized control of the cross-linking degree enabled by intensity-controlled photopolymerization. The UV dose can be adjusted with a photomask, where the cross-linking degree is proportional to the transmittance. For higher UV doses, invisible patterns are obtained, which were only revealed under deformation. For lower UV doses, optical patterns are already visible even in the rest state. In both cases, the cross-linking degree is related to the Poisson’s ratio of the CLCE, and nonuniform thickness changes when the label is submitted to mechanical deformation can be programmed. A nonuniform thickness change might lead to a mismatch between the spectral blue shift of the pattern and the surrounding background, resulting in the color-separation effect. We believe that these patterned mechanochromic labels may be particularly useful in stress–strain sensors, building environments, smart clothing, and anticounterfeiting labels, but they also open new opportunities for the fashion industry and art design.

## Supporting Information Available

Molecular structure of the chemicals; ^1^H NMR characterization and DSC thermogram of the oligomer; DSC thermograms and FTIR spectra of the CLCEs cross-linked with different UV doses; optical micrograph of the cross-section of the labeled fabric; POM images of the CLCEs acquired under different temperatures and additional pictures of the samples (PDF)
Stress test—UV cross-linked with 4 mJ/cm^2^ label (MP4)
Uniaxial strain test—UV cross-linked with 9 mJ/cm^2^ label(MP4)
Latex glove —UV cross-linked with 4 mJ/cm^2^ label (MP4)
Atom icon label—UV cross-linked with pattern: 4 mJ/cm^2^ and background: 6 mJ/cm^2^ (MP4)
Emoji icon label—UV cross-linked with pattern: 3 mJ/cm^2^ and background: 6 mJ/cm^2^ (MP4)
Atom icon label heated at 120 °C—UV cross-linked with pattern: 4 mJ/cm^2^ background: 6 mJ/cm^2^ (MP4)
