# Supplementary material for: Sticky Multicolor Mechanochromic Labels
Source: ACS Appl Mater Interfaces. 2024 Mar 6;16(11):14144–51. doi: 10.1021/acsami.3c19420 (PMC10958449; doi:10.1021/acsami.3c19420)
Supplement: Supplementary file 1 — am3c19420_si_001.pdf [file am3c19420_si_001.pdf]

Supporting Information

STICKY MULTICOLOR MECHANOCHROMIC LABELS

Lucas D. C. de Castro[1,2], Tom A. P. Engels[3], Osvaldo N. Oliveira Jr*[,1], and Albert P. H. J. Schenning[*,2,4]

[1]São Carlos Institute of Physics, University of São Paulo, São Carlos, SP, Brazil

[2]Laboratory of Stimuli-responsive Functional Materials and Devices (SFD), Department of Chemical Engineering and Chemistry, Eindhoven University of Technology, Eindhoven, The Netherlands

[3]Processing and Performance of Materials, Department of Mechanical Engineering, Eindhoven University of Technology, Eindhoven, The Netherlands

[4]Institute for Complex Molecular Systems, Eindhoven University of Technology, Eindhoven, The Netherlands

Corresponding authors: chu@ifsc.usp.br and a.p.h.j.schenning@tue.nl

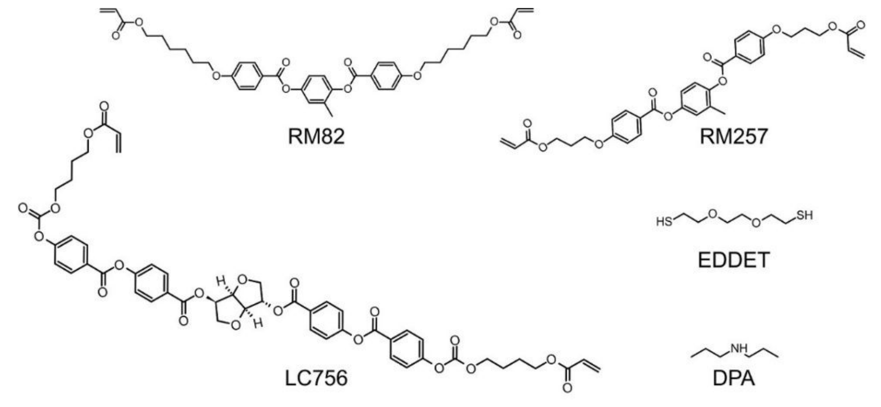

Figure S1. Molecular structure of the chemicals used for preparing the CLC acrylate-terminated oligomer ink.

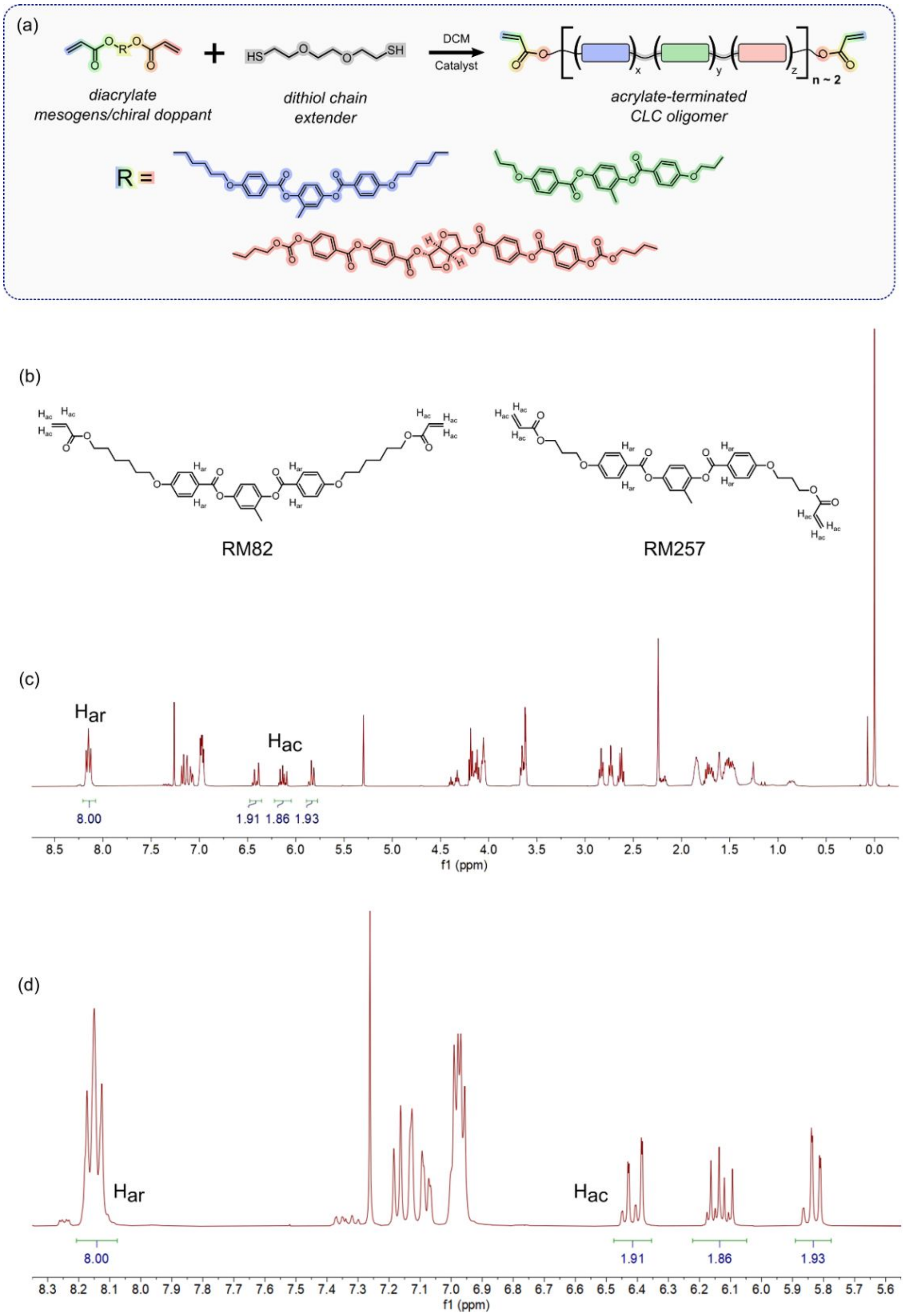

Figure S2. ^{1}H-nuclear magnetic resonance (^{1}H-NMR) characterization of the oligomer. (a) Schematic illustration of the structure of the oligomer. (b) Chemical structures of monomers RM82 and RM257. (c) Full ^{1}H-NMR spectrum. (d) Relevant part of the ^{1}H-NMR spectrum used for the calculation of the average degree of polymerization (DP). The DP can be determined by the ratio between the aromatic hydrogens (H_{ar}) and reactive acrylate hydrogens (H_{ac}) according to $\mathrm{DP} = (3/2) * \left(\int \mathrm{H_{ar}} / \int \mathrm{H_{ac}}\right)$.

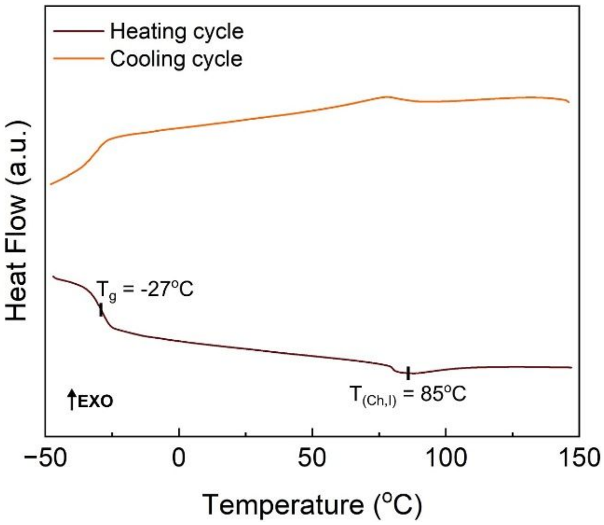

Figure S3. DSC thermograms of the CLC monomer mixture recorded during the second heating and cooling cycles. The glass transition temperature (T_g) and cholesteric to isotropic transition temperature ($T_{Ch,I}$) were obtained from the heating cycle.

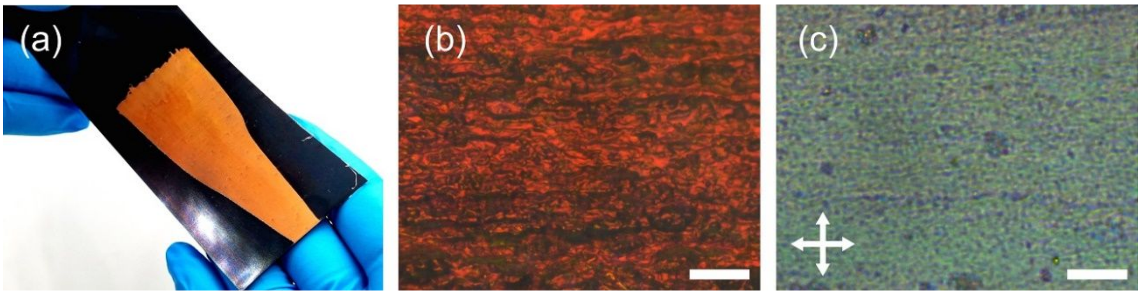

Figure S4. Shear-induced planar alignment of the CLCE. (a) Picture of the CLCE deposited via bar coating on the PET/PVA substrate after the crosslinking step with 4 mJ/cm² (scale bar is 2 cm). POM images of the respective CLCE in (b) reflection mode without analyzer and polarizer; and (c) transmission mode with crossed linear polarizers (scale bars are 25 μm). For the image acquisition on transmission mode, the black PET substrate was replaced by a similar transparent PET film and the CLCE was deposited on the same conditions.

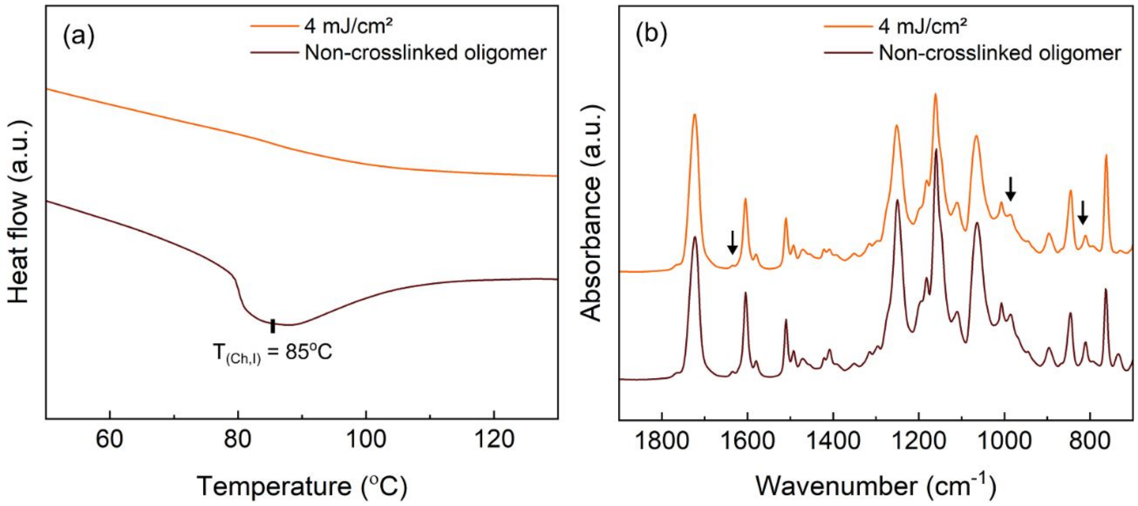

Figure S5. Thermal and chemical properties of the CLCE sticker UV crosslinked with 4 mJ/cm². (a) DSC thermograms and (b) FTIR spectra of the non-crosslinked oligomer and posterior to the UV crosslinking. The black arrows indicate the presence of non-reacted acrylate groups (1635; 985; and 812 cm^{-1}).

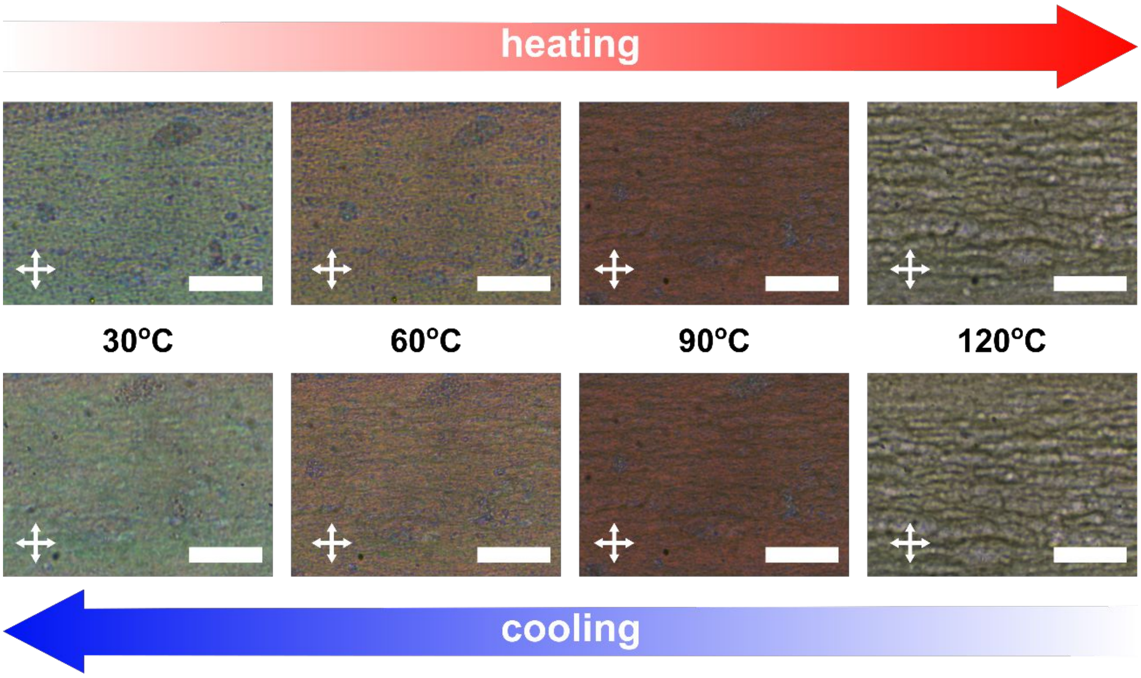

Figure S6. POM images of the CLCE crosslinked with 4 mJ/cm² acquired in transmission mode with crossed linear polarizers during heating and cooling cycles from 30°C to 120°C (scale bars are 50 μm). Although no discernible $T_{Ch,I}$ peak could be identified on the DSC thermogram of the sample UV crosslinked with 4 mJ/cm², a cholesteric to isotropic transition is observed by polarized optical microscopy (POM) at around 120°C. Due to the elastomeric behavior of the CLCE, the alignment is fully recovered when the system is cooled down.

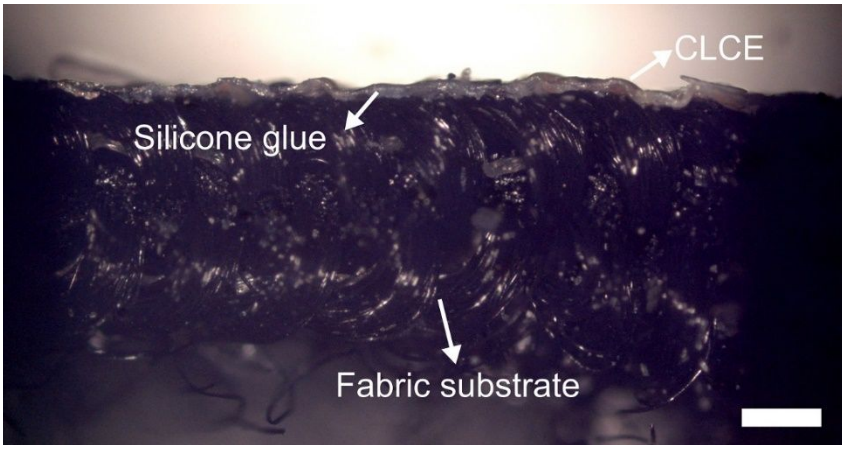

Figure S7. Optical micrograph of the cross-section of the labeled fabric. The labeled fabric is composed by a thin CLCE layer sticked to a fabric substrate with silicone glue. The thickness of the substrate, silicone glue and CLCE are respectively around 750 µm, 40 µm and 16 µm (scale bar is 250 µm).

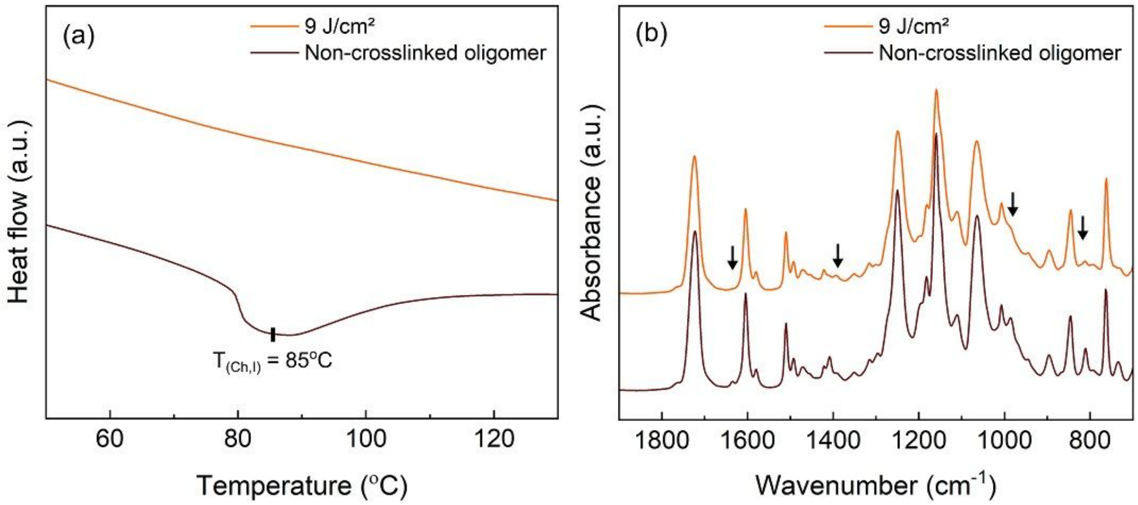

Figure S8. Thermal and chemical properties of the CLCE sticker UV crosslinked with 9 J/cm². (a) DSC thermograms and (b) FTIR spectra of the non-crosslinked oligomer and posterior to the UV crosslinking. The black arrows indicate the absence of acrylate groups (1635; 1407; 985; and 812 cm^{-1}).

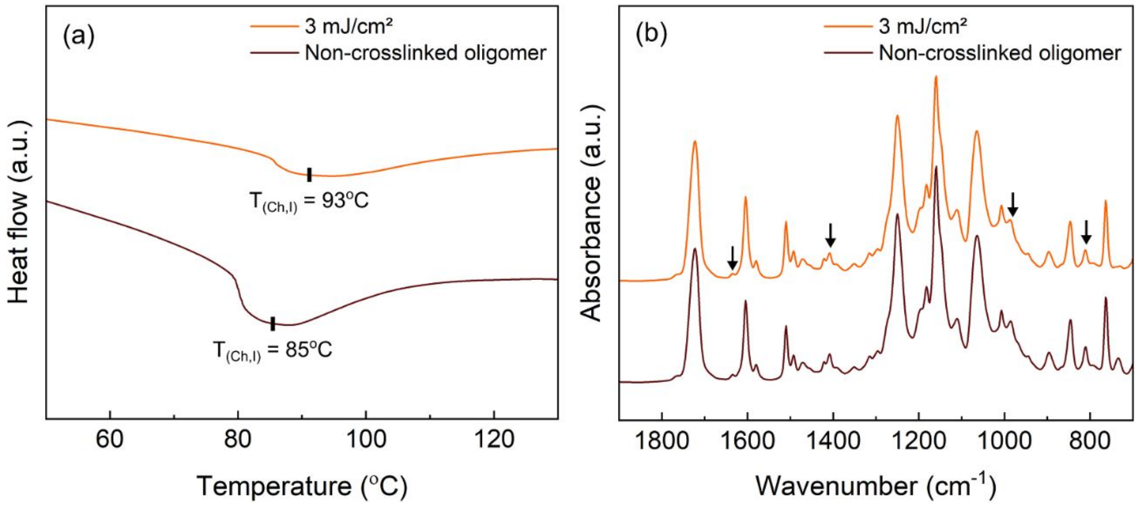

Figure S9. Thermal and chemical properties of the CLCE sticker UV crosslinked with 3 mJ/cm². (a) DSC thermograms and (b) FTIR spectra of the non-crosslinked oligomer and posterior to the UV crosslinking. The black arrows indicate the presence of non-reacted acrylate groups (1635; 1407; 985; and 812 cm^{-1}).

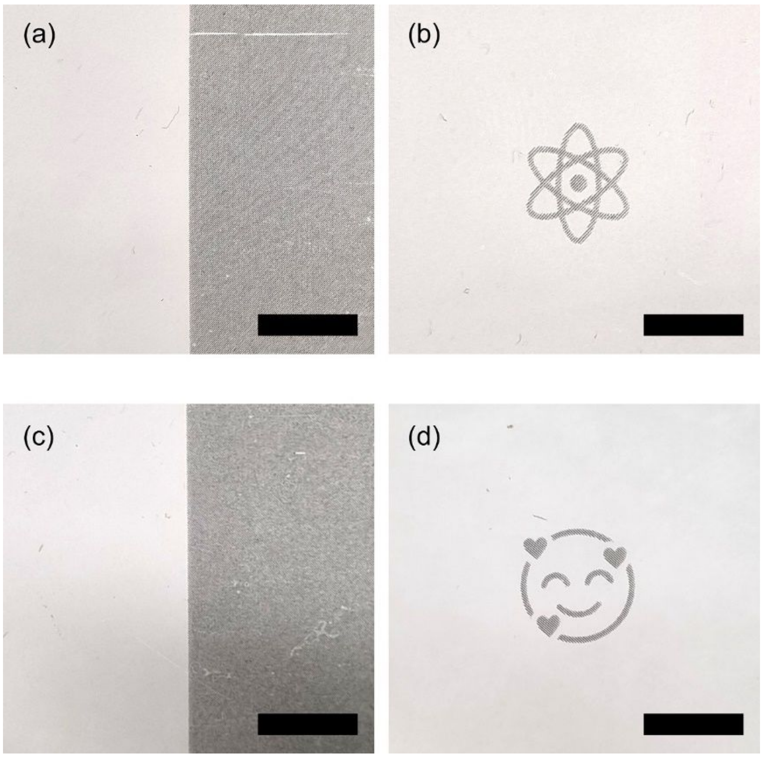

Figure S10. Pictures of the photomasks prepared on a transparent polyester film using a standard laser printer and placed between the light source and the sample during the UV crosslinking step. (a) bipartite pattern adjusted to be 6 and 4 mJ/cm² in the clear and gray regions, respectively; (b) atom icon; (d) bipartite pattern adjusted to be 6 and 3 mJ/cm² in the clear and gray regions, respectively; and (d) emoji icon (scale bars are 1 cm).

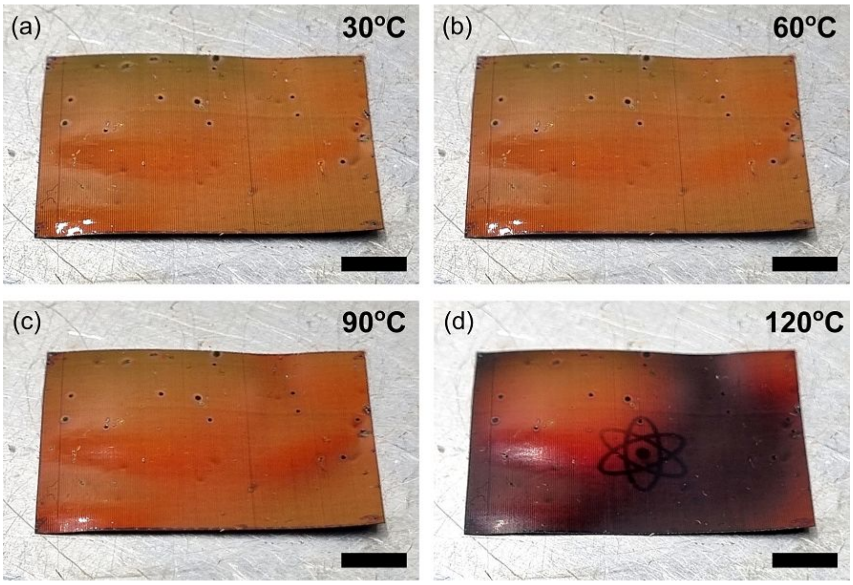

Figure S11. Pictures of the CLCE imprinted with an atom icon (pattern: 4mJ/cm² and background: 6 mJ/cm²) submitted to different temperatures: (a) 30°C; (b) 60°C; (c) 90°C; and (d) 120°C (scale bars are 1 cm). As the sample imprinted with an atom icon (pattern: 4mJ/cm² and background: 6 mJ/cm²) is heated until 120°C, a contrast between the pattern and the background arises and the atom icon becomes clearly visible. Immediately after the removal of the heating source, the system returns to the initial state and the atom icon becomes invisible again. This process is fully reversible as shown in **Movie S6.**
